# Changes in expression of Class 3 Semaphorins and their receptors during development of the rat retina and superior colliculus

**DOI:** 10.1186/s12861-014-0034-9

**Published:** 2014-07-26

**Authors:** Anil Sharma, Chrisna J LeVaillant, Giles W Plant, Alan R Harvey

**Affiliations:** 1 School of Anatomy, Physiology and Human Biology, The University of Western Australia, 35 Stirling Highway, Crawley 6009, WA, Australia; 2 Stanford Partnership for Spinal Cord Injury and Repair, Department of Neurosurgery, School of Medicine, Stanford University, Lorry I. Lokey Stem Cell Research Building (SIM1), 265 Campus Drive, Stanford 94305-5454, CA, USA

**Keywords:** Retinal ganglion cells, Collapse assay, qPCR, Neuropilins, Plexins, Cell adhesion molecules

## Abstract

**Background:**

Members of the Semaphorin 3 family (Sema3s) influence the development of the central nervous system, and some are implicated in regulating aspects of visual system development. However, we lack information about the timing of expression of the Sema3s with respect to different developmental epochs in the mammalian visual system. In this time-course study in the rat, we document for the first time changes in the expression of RNAs for the majority of Class 3 Semaphorins (Sema3s) and their receptor components during the development of the rat retina and superior colliculus (SC).

**Results:**

During retinal development, transcript levels changed for all of the Sema3s examined, as well as *Nrp2*, *Plxna2*, *Plxna3*, and *Plxna4a*. In the SC there were also changes in transcript levels for all Sema3s tested, as well as *Nrp1*, *Nrp2*, *Plxna1*, *Plxna2*, *Plxna3*, and *Plxna4a*. These changes correlate with well-established epochs, and our data suggest that the Sema3s could influence retinal ganglion cell (RGC) apoptosis, patterning and connectivity in the maturing retina and SC, and perhaps guidance of RGC and cortical axons in the SC. Functionally we found that SEMA3A, SEMA3C, SEMA3E, and SEMA3F proteins collapsed purified postnatal day 1 RGC growth cones *in vitro*. Significantly this is a developmental stage when RGCs are growing into and within the SC and are exposed to Sema3 ligands.

**Conclusion:**

These new data describing the overall temporal regulation of Sema3 expression in the rat retina and SC provide a platform for further work characterising the functional impact of these proteins on the development and maturation of mammalian visual pathways.

## Background

The mammalian visual system is complex, and the developmental events that give rise to this highly organised system are similarly complex and exquisitely ordered. The timing of the major developmental events of the mammalian visual system has been well characterised, and many of the molecular cues controlling these critical events have been elucidated (for example Ephs/Ephrins, Slits/Robo, Netrin/DCC, and various neurotrophic factors [1]–[7]). However despite our increased knowledge about these molecular cues, they are not sufficient to explain completely the complexity and timing of the development of visual pathways in mammals. It is likely then that other molecules are also involved during the development and maturation of the mammalian visual system, and in this context recent attention has turned to the Semaphorins.

Semaphorins (Semas) have been implicated in neural and vascular aspects of visual system development in various species including frog [8],[9], zebrafish [10]–[14], goldfish [15], and chicken [16]–[21]. In mouse the membrane bound Class 5 and Class 6 Semaphorins are involved in lamination of the retina [22]–[24], and guidance of retinal ganglion cell (RGC) axons [25] via contact-mediated interactions. However many previously discovered molecular cues in the mammalian visual system are diffusible, and the only vertebrate secreted members of the Semaphorins are the Class 3 Semaphorins (Sema3s) [26] which are known to be expressed in the developing rat retina [27]. It is possible then that the Sema3s also influence the complexity and timing of the developing mammalian visual system.

The Sema3s consist of *Sema3a* through *Sema3g*[16],[26],[28]–[36] and their main receptors are the Neuropilins which form multimeric receptor complexes with Plexins and cell adhesion molecules [37]. Sema3s were initially discovered as axon guidance molecules [30],[38], but are now known to also mediate apoptosis, cell migration, immune response, organogenesis, tumour suppression and promotion, and vasculature development [39]–[44]. Sema3s affect at least some aspects of the development of the visual system of rodents [45],[46], from where much of our knowledge of molecular and activity-driven influences on mammalian visual system development has come [6],[47].

**Figure 1:**
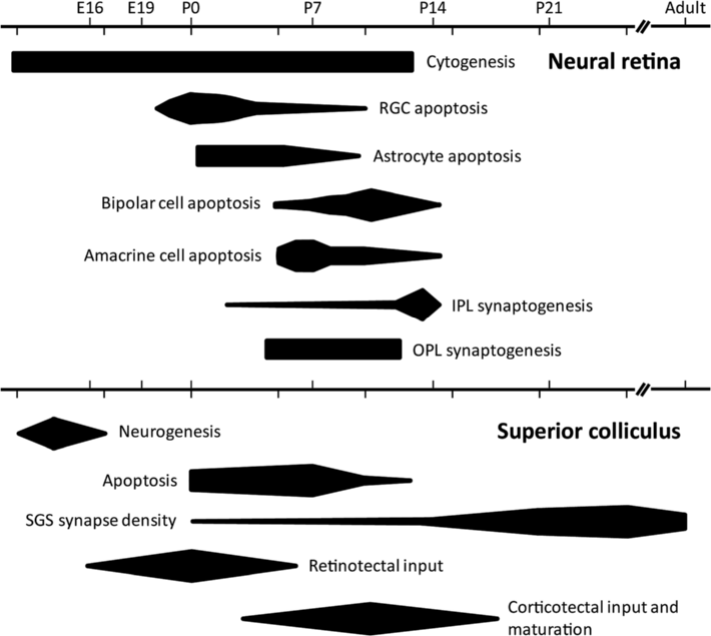
**Developmental epochs in the rat neural retina and superior colliculus.** Cytogenesis in the rat retina occurs from E9 through to P12, with distinct and overlapping waves of cell birth and differentiation [48]–[51]. Death of differentiated retinal ganglion cells (RGCs) is predominantly perinatal; starting at E20, peaking between birth and P3, falling sharply at P5 and continuing on until around P10 [52]–[57]. Astrocyte apoptosis is observed from P0, peaking between then and P5, and is associated with vasculature development [51],[58]. Bipolar and amacrine cell apoptosis occurs concurrently with different peaks, from P5 until P13/P14 [54]. Synapse generation in the inner plexiform layer (IPL) starts at P2 with amacrine cells [59],[60], and from P12 in bipolar cells [59], both peaking around the time of eye opening [54],[59]. Outer plexiform layer (OPL) synaptogenesis begins at P4 [59]–[62] and, while its time-course in the rat is not certain, in the mouse continues until about P12 [63]. Neurogenesis in the rat superior colliculus (SC) occurs from E12 to E17 [64],[65]. Apoptosis in the visual layers of the rat SC (stratum griseum superficiale; SGS) occurs from birth, peaking at between P4 and P7, and lasting until P13 [66],[67]. Circuit maturation in the SGS, as judged by synapse density and composition, is mostly postnatal [68],[69] in three stages: P0-P12/P14 (eye opening), P12-P24 increasing number of synapses, P21-P30/P40 increasing ratio of inhibitory to excitatory terminals [70]. Retinal axons first enter the SC at E16/E17 [71], and continue to arrive until P5/P6 [52],[72]. Cortico-tectal innervation starts at P3, with marked ingrowth between P6 and P12, and adult-like innervation observable from P18 [73]. Scale: downwards ticks mark 5 days, upwards ticks mark time points in this study; IPL: inner plexiform layer; OPL: outer plexiform layer; RGC: retinal ganglion cell; SGS: stratum griseum superficiale.

In summary, while we understand much about the timing of developmental events in the mammalian visual system, investigation of a potential role for the Sema3s in these events would benefit from knowledge about the timing of expression of Sema3s and their receptors during visual system maturation. We sought to address this gap in our knowledge by building on a previous study from our laboratory [27], focusing on Sema3s known to be expressed in RGCs and extending the work to include a greater number of receptors as well as analysis of a major central target for those RGCs, the superior colliculus (SC). To that end, we quantified transcript expression in the rat retina and SC over a range of embryonic (E) and postnatal (P) developmental time points (E16, E19, birth – P0, P7, P14, P21 and adult) which were chosen to correspond to previously established developmental epochs such as the timing of cell birth, migration, naturally occurring cell death, axonal and dendritic growth, and synaptogenesis (Figure 1). We also investigated the hypothesis that Sema3 ligands in the SC can influence RGC axon guidance using an *in vitro* growth cone collapse assay on isolated RGCs from a developmental stage when they would be growing into and within the SC *in vivo*.

## Results

**Figure 2:**
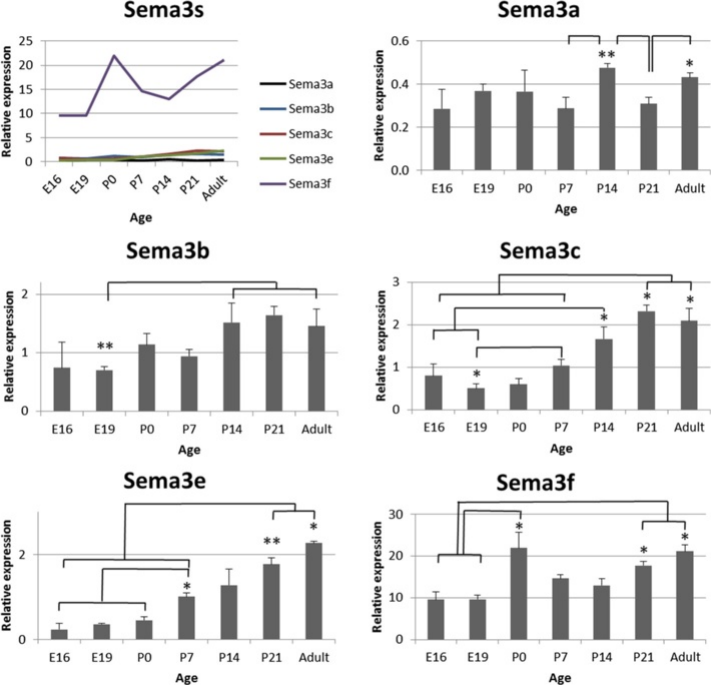
**RNA expression levels of the Class 3 Semaphorins in the rat retina during development.** Sema3s: *Sema3f* is the most predominant transcript, and *Sema3a* the least. All transcripts increased expression through development and maintained relatively high levels into adulthood. Sema3a: Expression rises slightly to a peak at P14, dropping at P21 before rising again at adulthood. Sema3b: Expression is lowest in the embryonic retina, around half that found from P14 onwards. Sema3c: Transcription is stable up to P0 then rises twofold by P21 and into adulthood. Sema3e: Expression levels rise steadily after birth, reaching fourfold higher levels at their peak in the adult. Sema3f: There are two periods of increased expression, a doubling at birth before falling again by P7 and then rising back by P21 and into adulthood. Error bars are SEM; * p < 0.05, ** p < 0.01; n = 4–5.

**Figure 3:**
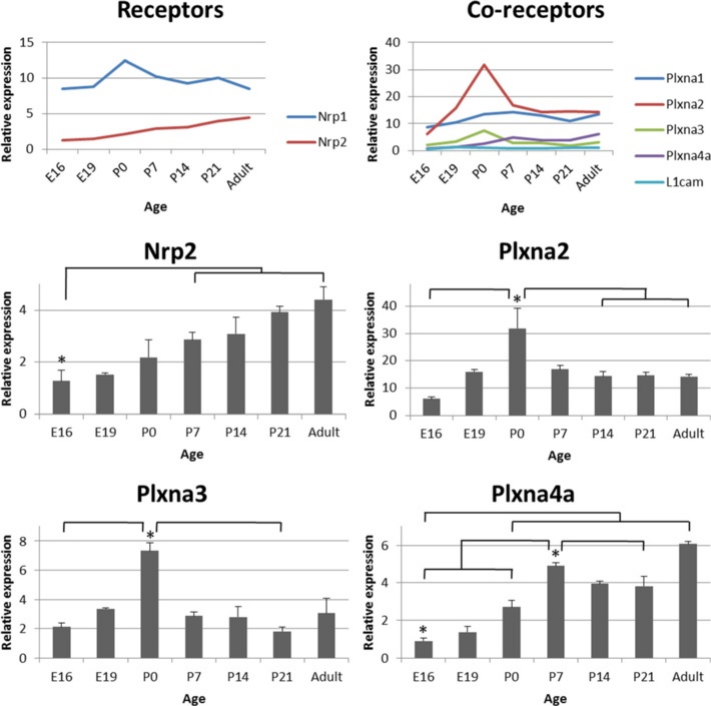
**RNA expression levels of the Class 3 Semaphorin receptors in the rat retina during development.** Receptors: *Nrp1* transcript levels are around twofold higher than the peak level of *Nrp2*. Co-receptors: *Plxna2* had the highest peak expression levels, and *L1cam* the lowest. *Plxna2* and *Plxna3* expression levels are very similar, with *Plxna2* having around fourfold higher peak levels. Nrp2: RNA levels are lowest in the embryo, rising from P7 onwards and peaking at around fourfold higher in the adult. Plxna2: Expression peaks at birth at fivefold higher than at E16, drops again by P14 and is maintained at that level into the adult. Plxna3: RNA levels increase fourfold from E16 to their peak at birth before falling back to embryonic levels by P21. Plxna4a: Expression triples from E16 to P0, doubles again at P7, falls back one third by P21 before increasing again to peak in the adult. Error bars are SEM;^*^ p < 0.05; n = 4–5.

**Figure 4:**
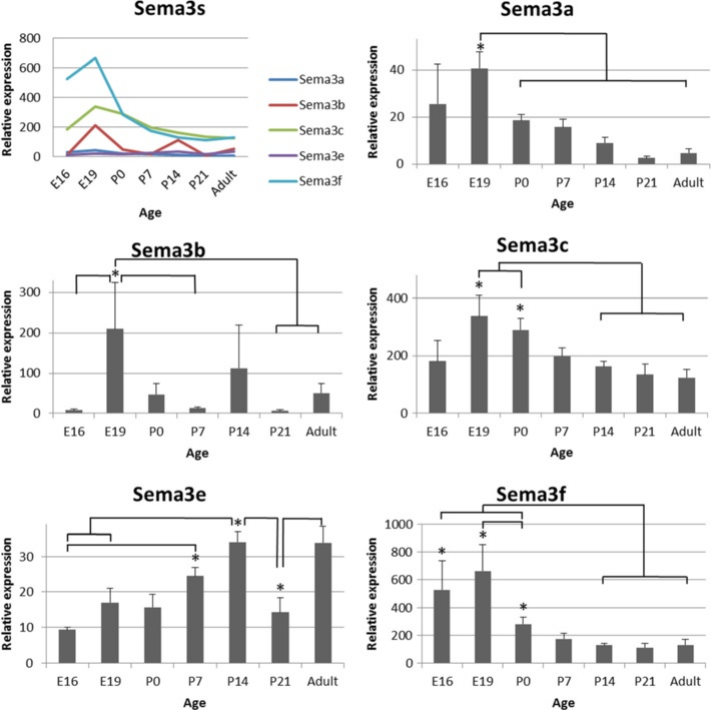
**RNA expression levels of the Class 3 Semaphorins in the rat superior colliculus during development.** Sema3s: All Sema3 RNA expression levels, except for *Sema3e*, peak at E19 and are lowest by P21 and into the adult. *Sema3b*, *Sema3c* and *Sema3f* are the predominant transcripts with around 100 times higher levels than *Sema3a* and *Sema3e*. Sema3a: There is a high variability in expression at E16, peak expression at E19, falling twofold by birth and eightfold in the adult. Sema3b: Expression changes markedly throughout development, with high variability levels at both E19 and P14. Expression rises twenty-eightfold from E16 to E19 before falling back 17 fold by P7 and back to E16 levels at P21. Transcript levels then rise eightfold from P21 to adult. Sema3c: RNA levels peak at E19 and P0, falling twofold by P14 and maintaining that level into the adult. Sema3e: Transcript levels double and triple from the embryo to P7 and P14 respectively, dropping back at P21 and increasing again into the adult. Sema3f: RNA expression levels are highest in the embryo, dropping fivefold after birth and into the adult. Error bars are SEM; * p < 0.05; n = 4–5.

**Figure 5:**
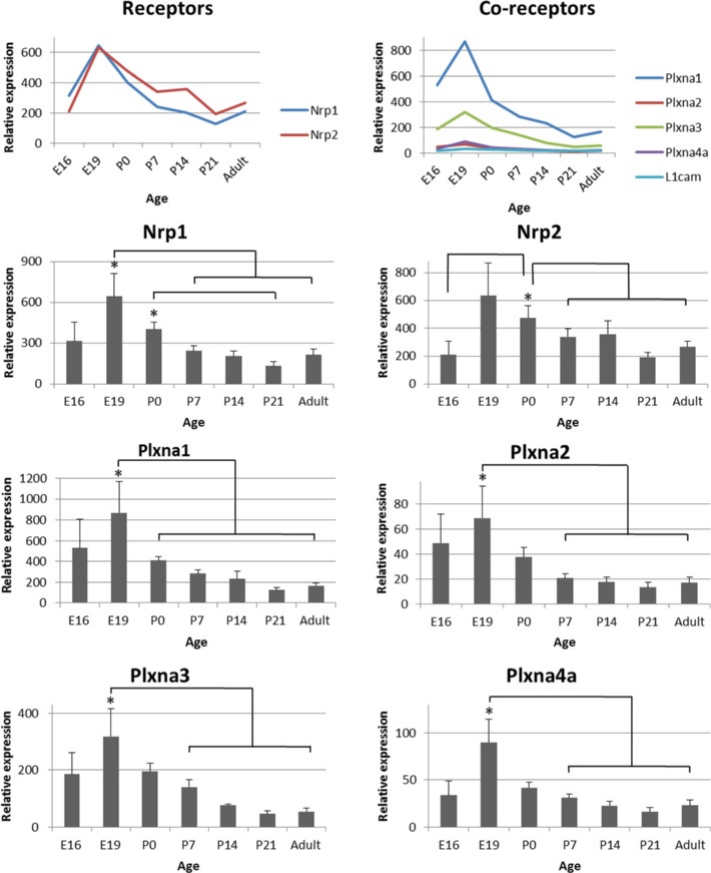
**RNA expression levels of the Class 3 Semaphorin receptors in the rat superior colliculus during development.** Receptors: *Nrp1* and *Nrp2* share similar expression profiles, with peaks around E19 and birth and then falling away into the adult. Co-receptors: All co-receptors bar *L1cam* follow the same expression profile, peaking at E19 before falling after birth and into the adult. *Plxna1* and *Plxna3* are the predominant transcripts. Nrp1: Peak RNA expression is at E19, falling threefold by P7 and maintained into the adult. Nrp2: Transcript levels double from E16 to P0, with high variability at E19. Expression falls from P0 to P7 and is maintained at around half peak levels in the adult. Plxna1: RNA levels are highly variable at E16 and E19 where they peak. From birth expression levels are halved, continue to decline up to P21 where they are sevenfold lower than at peak, and this level is maintained into the adult. Plxna2: RNA levels are highly variable at E16 and E19 where they peak. Expression drops threefold by P7 and is maintained into the adult. Plxna3: RNA levels are highly variable at E16 and E19 where they peak. Expression falls to half peak at P7, one quarter peak at P14 and is sevenfold lower than peak by P21 and in the adult. Plxna4a: RNA levels are highly variable at E16 and E19 where they peak before falling fourfold by P7 and into the adult. Error bars are SEM; * p < 0.05; n = 4–5.

**Figure 6:**
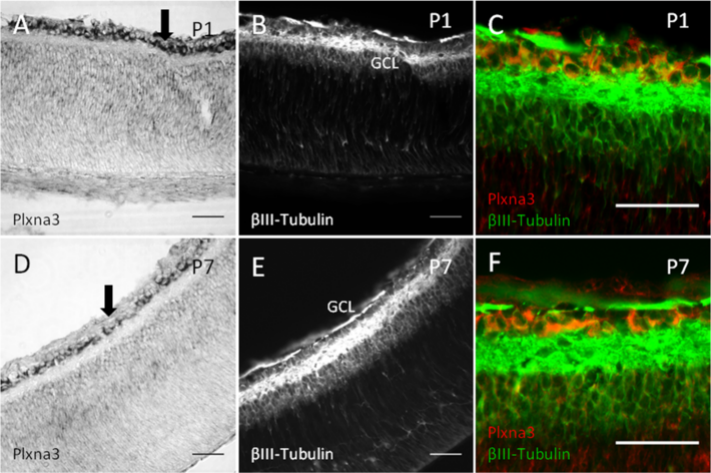
**Location of** ***Plxna3*** **transcript in the neonate rat retina.** Double *in situ* hybridisation (ISH) and immunofluorescence staining in P1 **(A-C)** and P7 retina **(D-F)**. Expression was restricted mainly to the ganglion cell layer (GCL), with overlap of *Plxna3* ISH and βIII-Tubulin positive putative retinal ganglion cells **(C,F)**. GCL and dark arrows: ganglion cell layer; scale bars: 50 μm.

**Figure 7:**
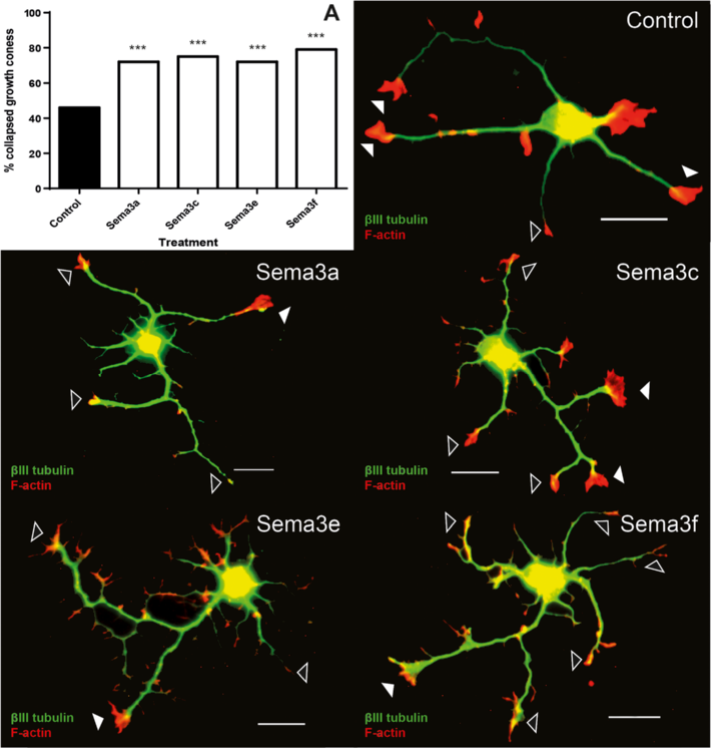
**Collapse assay of immunopurified P1/P2 rat retinal ganglion cells exposed to exogenous recombinant Sema3 proteins.** P1 retinal ganglion cells (RGCs) were immunopurified and seeded in BD Culture Slides coated with PDL and laminin, grown overnight. The following day (effectively P2), RGCs were exposed to recombinant Sema3s for 30 minutes before fixation and immunostaining. Growth cones were considered collapsed if the F-actin did not extend past the end of the βIII-tubulin positive neurite with an arc of less approximately 60°. Only terminal growth cones were counted, because lateral extensions can occur in response to the collapse of the leading growth cone. Additionally, growth cones were only scored if their neurites were at least two soma widths long. All cultures treated with conditioned media containing recombinant Sema3 proteins showed statistically significantly higher numbers of collapsed growth cones when compared to cultures treated with control conditioned media **(A)**. Also shown here are examples of neurons from each culture condition with growth cones scored as collapsed (hollow arrows) and uncollapsed (solid arrows). Solid arrows: uncollapsed growth cones; hollow arrows: collapsed growth cones; *** p < 0.001; scale bar: 20 μM.

Developmental expression profiles for the Sema3s and their receptors are presented in Figures 2, 3, 4 and 5, with those showing statistically significant changes in expression explored further. Statistical comparisons illustrated in Figures 2, 3, 4 and 5 are summaries of statistically significant peaks in expression; the entirety of statistical comparisons can be found in Additional file 1: Table S1. *Plxna3 in situ* hybridisations at P1 and P7 are presented in Figure 6 showing co-expression in βIII-tubulin positive cells (presumptive RGCs). The effects of exogenous recombinant Sema3s on RGC growth cone collapse are presented in Figure 7, and data detailing expression and detection of those recombinant Sema3s are presented in Additional file 2: Figure S1.

### Changes in Class 3 Semaphorins and their receptor components during rat retinal development

Expression levels in the retina could be separated into three qualitative groups: relatively high expression of *Sema3f* and *Plxna2*; moderate expression of *Nrp1* and *Plxna1*; and relatively low expression of the rest (Figures 2, and 3). There were statistically significant changes in the level of expression of all Sema3 RNAs in the retina, while of the receptors only *Nrp2*, *Plxna2*, *Plxna3*, and *Plxna4a* showed statistically significant changes (Figures 2, and 3; Additional file 1: Table S1).

Relative to other time points, *Sema3a* transcript expression levels were significantly increased at P14 and in the adult. Similar to *Sema3a*, *Sema3b* transcript expression was relatively stable during retinal development until P14, at which time there was increased expression that was maintained into adulthood. *Sema3c* RNA expression was also relatively steady through to P0, increasing significantly through to P21, and remaining at that level into the adult. *Sema3e* RNA levels appeared to increase gradually with retinal maturation and were significantly higher than E16-P7 levels at P21 and in adult rats. *Sema3f* transcription was temporarily greater at P0 and then increased again at P21 and beyond. *Nrp2* RNA expression gradually increased throughout retina maturation, levels at P7 and beyond being significantly greater than in embryos. *Plxna2* and *Plxna3* showed nearly identical patterns of altered transcript expression during development, both peaking significantly at the time of birth (P0). *Plxna4a* RNA levels peaked later at P7, but also again in the adult.

Many of the significant peaks in transcript expression occurred after the main developmental epochs. However, the changes that were quantified in the retina before P21 occurred during periods of RGC apoptosis, and synapse generation and maturation.

### Changes in Class 3 Semaphorins and their receptor components during development of the rat superior colliculus

There were four qualitative groups of transcript expression levels in the SC: *Plxna1* having the highest; followed by *Sema3f*, *Nrp1*, and *Nrp2*; then *Sema3b*, *Sema3c* and *Plxna3*; and lower amounts for the remainder (Figures 4, and 5). With the exception of *L1cam*, all genes of interest changed expression significantly during SC maturation.

*Sema3a* RNA levels were highly variable at E16 (149% coefficient of variance; CV), peaked at E19, and a decline with increasing postnatal age. The expression profile of *Sema3b* differed from other transcripts; there was large biological variation at many time points, with CVs greater than 100%, and four apparent peaks in expression, only one of which reached significance (E19). *Sema3c* transcript expression peaked at E19 and P0, before falling to adult levels by around P14. In contrast to the other Sema3s, *Sema3e* transcript levels increased steadily from E16 to P14, before a decrease at P21, then again increased in the adult. *Sema3f* expression was highest at E16 and E19, with RNA levels lower at birth and remaining at those reduced levels during postnatal development. There was relatively high biological variation in expression levels of *Nrp1*, *Nrp2* and all PlexinAs at E16 and E19 (CV > 100%) compared to other time-points. *Nrp1* RNA expression peaked at E19 then decreased steadily to P7 at levels that were maintained into the adult. *Nrp2* transcript levels peaked at E19 before falling steadily to E16 levels at P7 and beyond. *Plxna1* RNA expression also peaked at E19, a pattern that was repeated for the remaining three PlexinA RNAs, all of which had significantly decreased RNA expression by P0 and P7 that remained at low levels into adulthood.

The patterns of expression of Sema3s in the developing SC were substantially different from those seen in the retina, with peak expression occurring around or before birth. Temporally, these changes in transcript expression occurred during the period of SC neurogenesis, synapse generation and maturation, and innervation of the SC by extrinsic afferents including those from the retina and visual cortex.

### Expression of *Plxna3* in the neonatal retina

Our qPCR data showed that peak *Plxna3* RNA expression in the retina occurred during the period of peak RGC apoptosis (Figure 3). While rat retinal cells expressing the Sema3 receptor components *Nrp1*, *Nrp2*, *Plxna1* and *Plxna*2 have previously been identified using ISH [74] there has been no such characterisation for *Plxna3*. We used ISH to examine the cellular location of *Plxna3* transcripts in P1 and P7 rat retinas (Figure 6) and found that *Plxna3* RNA was clearly expressed in the ganglion cell layer at both these ages, with transcript expression overlapping βIII-tubulin (RGCs) immunostaining. This is in agreement with previously published data in the mouse retina [22]. Qualitatively, changes in the level of *Plxna3* expression in our ISH data are consistent with the reduction in expression between P0 and P7 in our qPCR material.

### Growth cone collapse assay for purified neonatal RGCS *in vitro*

It has been suggested that Sema3s in the SC can assist in the guidance of ingrowing RGC axons [45], and our data revealed increased expression of these ligands in the SC during this period (Figure 4). To further investigate the capability of Sema3s to influence RGC axons as they grow into and within the SC we used a growth cone collapse assay to assess the capacity of developmentally appropriate RGCs to respond to Sema3 proteins. Recombinant Sema3 proteins (SEMA3A-GFP, SEMA3C-FLAG, SEMA3E-FLAG, and SEMA3F-AP) in conditioned media were used to challenge immunopurified P1/P2 RGCs *in vitro*. These proteins were detected in the conditioned media by western blot against their artificial epitopes (Additional file 2: Figure S1A). Detected bands for SEMA3A-GFP correspond to the approximately 130 kDa and 90 kDa bands reported previously [75]. SEMA3C-FLAG was detected at around 80 kDa as expected, and also in a presumably processed form at around 70 kDa. Both SEMA3C-FLAG and SEMA3E-FLAG were detected in media at approximately 100 kDa, which might represent glycosylated forms of the proteins. SEMA3F-AP was detected at 150 kDa, in line with expectations. Transfected cells were analysed in culture by epifluorescence and immunocytochemistry and were positive for recombinant proteins while appropriate controls were negative (Additional file 2: Figure S1B-G).

Growth cones were visible as F-actin positive extensions from the ends of βIII-tubulin positive neurites. Typically uncollapsed growth cones displayed the common ‘hand like’ morphology, and collapsed growth cones appeared as thin stumps. Growth cones were counted only at the end of neurites, even when significant growth cone-like processes were observed along the shaft of a neurite, as these lateral extensions can occur in response to the collapse of the leading growth cone [76]. Examples of collapsed versus uncollapsed growth cones are illustrated in Figure 7. Cultures treated with conditioned media containing any of the Sema3s had significantly higher percentages of collapsed growth cones, with the average percentage of the two replicates and statistical significance level presented in Figure 7A. Our data demonstrate that neonatal rat RGCs are competent to respond to SEMA3A, SEMA3C, SEMA3E, and SEMA3F proteins at a developmental time point during which their axons are entering into and growing within the SC *in vivo*[52],[71],[72].

## Discussion

We found statistically significant changes in gene expression of Sema3s and their co-receptors during maturation of the rat retina and SC. These changes differed in degree and time-course between the retina and SC so were not due to generalised or non-specific systemic changes. It is likely that the observed tissue-specific changes in expression reflect the biological roles of these molecules and are in some way related to the development and maturation of each structure [20],[21],[45]. While our data cannot localise these changes in expression to distinct cell populations, previous work in the retina suggests that the transcripts studied are heavily expressed in rat RGCs [27]. Importantly we also showed that neonatal rat RGCs are competent to respond to Sema3s at a developmental time point when their axons are encountering the Sema3s in the SC. We are wary of over-analysing temporal association data, but to help place our results in context we briefly discuss our data below with respect to known developmental processes (Figure 1).

### Class 3 Semaphorins and morphogenesis of the retina and superior colliculus

Sema5s and Sema6s are known to mediate lamination of the mouse retina, signalling through *Plxna2*, *Plxna3* and *Plxna4*[22]–[24]. In addition, Sema3s guide cells as they differentiate and migrate within CNS tissues such as the cortex, cerebellum, and hippocampus [39],[77],[78], and presumably they are able diffuse through tissues like other guidance cues in the mammalian visual system such as the Ephs and Netrin. Is the expression of Sema3s also involved in morphogenesis of the rat retina and SC? Retinal neurogenesis occurs from E10/E11 through to the second postnatal week [48], and while there is expression of all Sema3s and their receptors and co-receptors during much of this period, expression levels are for the most part relatively low. Rod photoreceptor generation peaks at P0, as does transcript expression of *Sema3f*, *Plxna2* and *Plxna3* RNAs. However there appears to be very little *Sema3f* transcript expression in the outer nuclear layer (ONL) at this age [27], despite evidence of relatively high expression of *Sema3f* RNA in the ONL of adult mice [79].

On the other hand, rat SC neurogenesis occurs from E12 to E17 [64],[65] and the SC has an adult like laminar structure at P7 [80]. *Sema3c* and *Nrp2* RNA expression displayed the same upwards then downwards sweep as neurogenesis in the SC: elevated at E19 and P0, falling by P7. Similarly, *Sema3a*, *Sema3b*, and *Sema3f* as well as *Nrp1* and all PlexinA co-receptors showed peak expressions at E19 that declined thereafter, coincidental with early cytoarchitectural patterning of the SC. These data therefore suggest that the Sema3s might play a role in patterning the SC, but not the retina.

### Class 3 Semaphorins and retinal ganglion cell apoptosis

Sema3s, specifically *Sema3a* and *Sema3f*, have been implicated in apoptosis of neurons including RGCs [81]–[86]. During retinal development, RGC apoptosis is predominantly perinatal [52]–[57] and we observed three genes that had clear peaks in retinal RNA expression at P0: *Sema3f*, *Plxna2*, and *Plxna3*. Interestingly, *Plxna2* and *Plxna3* are co-receptors for *Sema3f* and *Sema3a*[37], and *Plxna3* has been shown to be directly involved in *Sema3a* mediated cell death [84]. *Sema3a*, *Sema3f*, and *Plxna2* are expressed in neonatal rat RGCs [27], and we here show that in the rat *Plxna3* RNA is predominantly expressed in the retinal ganglion cell layer (presumptive RGCs) at P1 (Figure 7). Importantly we also showed that P1/P2 rat RGCs were responsive to both *Sema3a* and *Sema3f* (Figure 7). Our data provide further suggestive evidence that the Sema3s may influence RGC apoptosis.

### Class 3 Semaphorin expression during the period of synapse generation and maturation in retina and superior colliculus

Sema3s are also involved in terminal axon branching, synaptogenesis, pruning of terminal arbors, regulation of synaptic plasticity, and maturation of dendrites [42],[87]–[92]. Synaptogenesis in the rat retina starts around P2 and is ongoing at P7 [54],[59]–[62], when transcripts for *Sema3c* and *Sema3e* both become more abundant. Conversely, *Sema3b* and *Sema3f* RNAs increased after completion of retinal synaptogenesis (P14) [63]. The maintained relatively high transcript levels in the adult rat retina of *Sema3b*, *Sema3c*, *Sema3e*, and *Sema3f* might indicate a role in regulating synaptic plasticity in the mature retina. It has been reported that *Plxna2*, *Plxna3*, and *Plxna4* are important for proper lamination of the inner plexiform layer (IPL) of the mouse retina, with mRNA expressed in amacrine cells and protein heavily expressed in the IPL [22]–[24]. We see no clear signal supporting this finding in the rat retina, although this may be a consequence of analysing global rather than cellular expression. In the SC, the majority of synaptogenesis is postnatal [68]–[70], and *Sema3e* RNA expression in the SC peaks during the period when many synapses are being formed (P14) suggesting a role for this molecule in SC synaptogenesis.

### Effect of Sema3s on guiding RGC and other axons into and within the superior colliculus

RGC axons enter the rat SC between E16 and P5/P6 [52],[71],[72], and previous results suggest that *Sema3f* may contribute in helping to guide these axons to their correct retinotopic targets [45]. Furthermore, the Sema3 co-receptor *L1cam* is required for correct mapping of retinal axons in the mouse [93],[94]. Previous data reveal that during development of the mouse SC at least some of the retino-tectal afferent fibres are positive for *Nrp1*, *Nrp2*, and *L1cam*[93]–[95], and P1 mouse RGC axons express the receptors *Nrp2* and *Plxna1*[45]. While previous studies have shown responsiveness of some rodent RGCs to some Sema3s [45], these were not conducted at a developmental time point where the RGC axons are growing into and within their Sema3 expressing targets [52],[71],[72]. Here we show for the first time that rat P1/P2 RGCs are capable of responding to exogenous Sema3 proteins at an age when many of these neurons are encountering these Sema3s as they grow axons into and within the SC. [52],[71],[72]. Our data show that almost all these Sema3s, bar *Sema3e*, had peak expression levels in the SC during this period of retinal afferent ingrowth, but that *Sema3c* and *Sema3f* had around tenfold higher expression compared to *Sema3a* and *Sema3e*. This difference in RNA expression levels might indicate that if these molecules are having an effect then the more likely candidates are *Sema3c* and *Sema3f*. However, some caution must be used when extrapolating levels of RNA expression to physiological significance; *L1cam*, *Plxna3* and *Plxna4a* all had relatively low expression in the retina despite their known effects in patterning the retina [22],[23],[93],[94]. Nevertheless, our data do support the suggestion that *Sema3f* may play a role in the formation of the retinotopic map in the mammalian SC [45].

Sema3s have also previously been shown to influence the pattern of long-distance cortico-tectal projections [96]. Rat visual cortical axons reach the SC at about P3, with marked ingrowth between P6 and P12, and then final maturation between P12-P18 [73]. Our data show elevated *Sema3e* expression during this period of ingrowth and maturation of cortical fibers in the SC, suggesting that *Sema3e* might influence the development of corticotectal projections.

## Conclusions

In conclusion, the analysis of the Sema3s and their co-receptors in the developing rat visual system revealed a number of new and potentially important findings. In the retina, mRNA expression levels changed for all Sema3s examined and there were age-specific changes in *Nrp2*, *Plxna2*, *Plxna3*, and *Plxna4a* mRNA expression. In the SC there were also maturational changes in transcript levels for all Sema3s, Neuropilins, and PlexinAs, although the time-course of these changes differed markedly from those seen in the retina. These developmental changes were associated with periods of RGC apoptosis; neurogenesis in the SC; synapse generation, maturation, and plasticity in the retina and SC; and innervation by retino- and cortico-tectal axons in the SC. Importantly, and consistent with a broad role for the Sema3 family in the maturing visual system, purified P1/P2 RGCs were sensitive to *Sema3a*, *Sema3c*, *Sema3e*, and *Sema3f* mediated growth cone collapse *in vitro*. These new data, providing a framework for future studies aimed at elucidating such roles of the Sema3s. These new data describing the overall temporal regulation of Sema3 expression in the rat retina and SC highlight maturational events that might be influenced by Sema3s, providing a platform for further work characterising the functional impact of these proteins on mammalian visual system development.

## Methods

Experiments conformed to the Australian National Health and Medical Research Council (NHMRC) guidelines and were approved by the Animal Ethics Committee of The University of Western Australia. Animals were sourced from the Animal Resources Centre (Western Australia). All statistical tests were performed using SPSS v19 (IBM, USA). E0 was the day vaginal plug detected, P0 was date of birth.

### Tissue collection

#### Tissue collection for qPCR

Embryonic Wistar rats aged E16 or E19 were dissected after caesarean section from mothers under halothane anaesthesia (5% (v/v) halothane (Rhone Merieux, Australia) in 80:20 N_2_O:O_2_) and immediately placed in ice-cold F-10 media (Gibco, Australia). Dams were sacrificed by intraperitoneal (IP) overdose (50 mg/100 g body weight) of pentobarbitone sodium (Lethabarb; Virbac, Australia). Postnatal and adult (8 to 10 week old) rats were anaesthetised by overdose of Lethabarb (IP, 32.5 mg/100 g body weight). Eyes and SCs were quickly dissected out and placed into fresh ice-cold F-10 media. Retinas were dissected free of the surrounding tunica and vitreous, left and right retinas pooled and placed immediately into RNA*later* (Ambion, USA). Similarly, SCs were dissected free of surrounding meninges and placed in RNA*later*. All tissues stored in RNA*later* were cooled to 4°C within one hour for overnight storage, before transfer into −20°C for longer term storage. Four to five animals were used per group.

#### Tissue collection for in situ hybridisation

P1 and P7 Wistar rats were anaesthetised by overdose injection of Lethabarb before transcardial perfusion with 0.05% (w/v) heparin (David Bull Laboratories, Australia) in phosphate buffered saline (PBS), followed by 4% paraformaldehyde (w/v; Sigma-Aldrich, Australia) in 0.1 M Sorenson’s Buffer (4% PFA; pH 7.4). Eyes and whole brains were dissected out and postfixed in 4% PFA for 30 minutes before cryoprotection in diethylpyrocarbonate (DEPC; Sigma-Aldrich, Australia) treated PBS containing 30% sucrose (w/v; Sigma-Aldrich, Australia) overnight at 4°C. PBS/sucrose solution was gradually replaced with Jung tissue freezing medium (Leica Microsystems, Australia) over several days. After infusion tissues were snap frozen in isopropanol (2-propanol; Sigma-Aldrich, Australia). 20 μM sections were cut on a cryostat, placed on SuperFrost Plus slides (Menzel-Gläser, Germany), and stored at −80°C until processing by *in situ* hybridisation (ISH).

### qPCR

Methodology for qPCR was as previously detailed [97], and briefly described here. Total RNA was extracted with Tri Reagent (Molecular Research Center, USA) and treated with recombinant DNase I (rDNase I; DNA-*free*; Ambion, USA). First strand cDNA was synthesised using Omniscript (Qiagen, Australia) and random hexamers (Promega, Australia). Previously validated primer pairs [97] were used to quantify RNA transcript expressions of *Sema3a*, *Sema3b*, *Sema3c*, *Sema3e*, *Sema3f*, *Plxna1*, *Plxna2*, *Plxna3*, *Plxna4a*, *Nrp1*, *Nrp2*, *L1cam*, and internal reference genes *Ppia*, *Rnr1*, and *Rpl19*. qPCR runs were performed on either a Rotor-Gene 3000 or 6000 (Qiagen, USA), using Bio-Rad iQ SYBR 2x Mastermix (Bio-Rad, Australia) in 10 μL reactions containing 500 nM of each primer, and C_q_ values obtained from using the inbuilt second derivative maximum (SDM) equation. qPCR efficiencies are averages of efficiencies calculated from individual reactions using LinRegPCR [98],[99]. Initial fluorescence levels (N_0_) were calculated from C_q_ and mean efficiency values [100], and then normalised to the geometric mean of appropriate internal reference genes [101]. BestKeeper analysis found all internal reference genes were appropriate internal controls, as judged by the standard deviation in C_q_ values. However, *Rpl19* was not used as an internal control because its RNA expression changed significantly between developmental groups (Kruskal-Wallis ANOVA, p < 0.05 and p < 0.01 in the retina and SC respectively), and was relatively poorly correlated to the BestKeeper index SC (Spearman’s R = 0.444). Thus expression data were normalised to the geometric mean of *Rnr1* and *Ppia* expression [101].

Normalised data were analysed for changes between developmental groups by Kruskal-Wallis ANOVA (analysis of variance). When Kruskal Wallis ANOVA resulted in p < 0.05 pairwise tests of statistical significance between time points were performed by Mann–Whitney *U* tests.

### *In situ* hybridisation

Plasmid for *Plxna3* riboprobe was kindly provided by Professor Joost Verhaagen (Netherlands Institute for Neuroscience, Amsterdam). Sense and anti-sense probes were produced by restriction endonuclease (RE; New England Biolabs, USA) digest followed by *in vitro* RNA transcription (IVT) with digoxigenin labelled RNA (Roche, Australia) and RNA polymerase (Roche, Australia). RE used were: sense, Kpn I; anti-sense, Spe I. Riboprobes were hydrolysed for one hour at 60°C, precipitated with LiCl and 100% ethanol, resuspended in DEPC treated double deionised water (DDW), and stored at −80°C before use.

P1 and P7 retinal sections were processed for ISH with the above riboprobe using methodology previously described [27], with α-dig-AP fragments (Roche, Australia) and NBT/BCIP (Roche, Australia). Sections were differentiated in 70% ethanol if necessary, and stored in PBS before proceeding to immunohistochemistry (IHC). Sense riboprobes produced limited, diffuse staining, and all experimental runs showed positive anti-sense riboprobe staining in control tissue (adult rat cerebellum; data not shown).

### Immunohistochemistry

After ISH processing, sections were processed for immunofluorescence using standard methods. Antibody diluent was 0.1% (w/v) Triton-X100, 10% (v/v) normal goat serum (NGS; Chemicon, USA), in PBS. Primary antibody (α-βIII-tubulin (TUJ1); mouse monoclonal, 1:2,000 dilution; Covance, USA) incubation was 4°C overnight in a humidified chamber, followed by secondary antibody (α-mouse FITC, goat raised; 1:400 dilution; ICN Cappel, USA) incubation for 2 hours in a dark humidified chamber at room temperature. Slides were coverslipped using fluorescent mounting media (DAKO, Australia).

Cell cultures were stained for βIII-tubulin and F-actin. βIII-tubulin was labelled by IHC according to standard practice, using the same antibody diluent, incubation times, and antibodies as described above. Following immunostaining for βIII-tubulin, cultures were incubated with phalloidin-FITC (Sigma-Aldrich, Australia) diluted 1:1,000 in PBS for 2 hours at room temperature to stain F-actin, then were washed 3 times with PBS, before being coverslipped using DAKO fluorescent mounting media.

### Recombinant Class 3 Semaphorin conditioned media

Expression plasmids (*Sema3a*-GFP, *Sema3c*-FLAG, *Sema3e*-FLAG, *Sema3f*-AP) were also a gift from Professor Joost Verhaagen. The *Sema3a*-GFP and *Sema3f*-AP plasmids have previously been characterised [75],[102], containing rat and mouse cDNA respectively. *Sema3c*-FLAG contains mouse *Sema3c* cDNA, and *Sema3e*-FLAG contains human *Sema3e* cDNA. Plasmid constructs were verified by sequencing.

HEK-293 T cultures were grown in DMEM: FBS (Dulbecco’s Modified Eagle Medium:fetal bovine serum; Gibco, Australia) 9:1 fortified with gentamycin (50 μg/mL; Invitrogen, Australia), and then grown without the presence of antibiotics overnight. Cells were transfected with plasmids using Lipofectamine 2000 (Invitrogen, Australia), using Opti-MEM (Invitrogen, Australia) as per manufacturer’s instructions. Media was changed to original growth media with antibiotics 6 hours after transfection, and conditioned media collected 30 hours later. Control conditioned medium was obtained by the same methods, using sham (no plasmid) transfected cultures.

### Immunoprecipitation and detection of recombinant proteins

SEMA3A-GFP was purified from conditioned media using magnetic GFP-Trap beads (Chromotek, Germany). FLAG tagged proteins (SEMA3C-FLAG and SEMA3E-FLAG), and Myc tagged proteins (SEMA3F-AP) were immunoprecipitated from conditioned media using Dynabeads Protein A/G (Invitrogen, Australia) and 5 μg α-FLAG antibody (Sigma-Aldrich, Australia), or 5 μg α-Myc antibody (CellSignaling, USA). Proteins were eluted in 2X Laemmili Buffer (250 mM Tris, 10% (v/v) glycerol, 4% (w/v) SDS, 2% (v/v) β-mercaptoethanol, 0.005% (w/v) bromophenol blue, in DDW pH 6.8), separated by SDS-PAGE (Mini-PROTEAN TGX Stain-Free Precast Gels; Bio-Rad, Australia) and transferred to nitrocellulose membrane (Trans-Blot Turbo Mini Nitrocellulose Transfer Pack; Bio-Rad, Australia). Recombinant proteins were detected by western blot using 5% (w/v) skim milk powder in Tris buffered saline containing Tween (TBS-T; 100 mM Tris, 154 mM NaCl, 0.1% (v/v) Tween 20, in DDW pH 7.5) blocking buffer, α-GFP antibody (mouse monoclonal clones 7.1 and 13.1, 0.4 μg/mL; Roche, Australia), α-FLAG antibody (mouse monoclonal, 10 μg/mL; Sigma-Aldrich, Australia), α-Myc antibody (mouse monoclonal, 1:1,000 dilution; Cell Signaling, USA), and α-mouse-HRP secondary antibody (1:10,000 dilution; Peirce Scientific, Australia). Proteins were visualised by chemiluminescence (Immun-Star; Bio-Rad, USA) using the Chemi-Doc system (Bio-Rad, USA).

### Immunopurified P1/P2 retinal ganglion cell cultures

P1 Wistar pups were euthanised by IP Lethabarb overdose and retinas dissected free in PBS. Retinas were dissociated with MiltenyiBiotec Tissue Dissociation Kit – Postnatal Neurons (MiltenyBiotec, Australia), and RGCs purified using the MACS RGC Isolation Kit (MiltenyiBiotec, Australia) according to manufacturer’s instructions. Purified RGCs were resuspended in growth media (Neurobasal media (Invitrogen, Australia), 1X B27 supplement (Invitrogen, Australia), 1.25 μM transferrin (Sigma-Aldrich, Australia), 0.2 μM progesterone (Sigma-Aldrich, Australia), 100 μM putrescine (Sigma-Aldrich, Australia), 230 nM sodium selenite (Sigma-Aldrich), 1.5 μM bovine serum albumin (Sigma-Aldrich, Australia), 872 nM bovine pancreas insulin (Sigma-Aldrich, Australia), 1 mM L-glutamine (Invitrogen, Australia), 30.6 μM N-*acetyl*-cysteine (Sigma-Aldrich, Australia), 6 nM triiodithyronine (T3; Sigma-Aldrich, Australia), 1 mM sodium pyruvate (Sigma-Aldrich, Australia)) fortified with growth factors (5 μM insulin (Sigma-Aldrich, Australia), 5 μM forskolin (Sigma-Aldrich, Australia), 440 pM rat ciliary neurotrophic factor (CNTF; PeproTech, USA), 1.85 nM human brain-derived neurotrophic factor (BDNF; PeproTech, USA) to increase survival as previously described [103]. Cells were seeded onto PDL (100 μg/mL; Sigma-Aldrich, USA) and mouse laminin (10 μg/mL; Invitrogen, Australia) coated BD CultureSlides (BD, Australia) at approximately 6,000 cells per mm^2^.

### Purified retinal ganglion cell growth cone collapse assay

Purified RGC cultures were grown overnight at 37°C and 5% pCO_2_. The following day (effectively P2) 30 μL of conditioned media was added to the 300 μL of growth media in each well and incubated at 37°C and 5% pCO_2_ for 30 minutes. Cultures were then fixed with 4% PFA and stained for βIII-tubulin and F-actin. Epifluorescent photomicrographs were taken of all neurons in all cultures, and encoded such that analysis was completed blind to treatment group. Growth cones were scored as either collapsed or uncollapsed [104]: uncollapsed if the neurite itself was at least two cell body widths in length, and the growth cone spread with an arc of at least approximately 60°; other growth cones on neurites at least two cell body widths in length were classified as collapsed. The number of collapsed and uncollapsed growth cones was normalised to totals in the control group, and compared using a Chi-square test. The minimum number of growth cones counted per group was 50, and the assay was repeated to confirm results.

## Competing interests

The authors declare that they have no competing interests.

## Authors’ contributions

AS carried out qPCR, in situ hybridisations, immunohistochemistry, data analyses, project coordination, manuscript preparation and revision. CL performed immunopreciptiation and western blotting. GWP participated in experimental design and manuscript revision. ARH conceived experimental design, aided in project management, and contributed heavily to manuscript revision. All authors read and approved the final manuscript.

## Additional files

## Supplementary Material

Additional file 1: Table S1. Pairwise statistical comparisons of expression data. Normalised data were analysed for changes between developmental groups by Kruskal-Wallis ANOVA (analysis of variance). When Kruskal Wallis ANOVA resulted in p < 0.05 pairwise tests of statistical significance between time points were performed by Mann–Whitney *U* tests. e: p = 0.05; * p < 0.05; ** p < 0.01. Click here for file

Additional file 2: Figure S1. Recombinant Sema3 protein expression in conditioned media. A: Presented are three different blots, lined up against the same ladder. Controls were from cultures that underwent a sham transfection (no plasmid). Boxes indicated bands of expected molecular weights. Detected bands for SEMA3A-GFP correspond to the approximately 130 kDa and 90 kDa bands reported previously [75]. SEMA3C-FLAG was detected at around 80 kDa as expected, and also in a presumably processed form at around 70 kDa. Both SEMA3C-FLAG and SEMA3E-FLAG were detected in media at approximately 100 kDa, which might represent glycosylated forms of the proteins. SEMA3F-AP was detected at 150 kDa, in line with expectations. B-G: recombinant Class 3 Semaphorin expression in HEK-293 T cells transfected with expression plasmids for *Sema3a*-GFP, *Sema3c*-FLAG, *Sema3e*-FLAG, and *Sema3f*-AP, as well as a no plasmid control. SEMA3A-GFP expression was visualised directly by epifluorescence (B), SEMA3B-FLAG (C) and SEMA3E-FLAG (D) was detected following fluorescence immunocytochemistry, and SEMA3F-AP was detected by the presence of colour reaction product from NBT/BCIP (F). Scale bar: 50 μM. Click here for file
