# Erratum to: Changes in expression of class 3 semaphorins and their receptors during development of the rat retina and superior colliculus

**DOI:** 10.1186/s12861-014-0039-4

**Published:** 2014-10-31

**Authors:** Anil Sharma, Chrisna J LeVaillant, Giles W Plant, Alan R Harvey

**Affiliations:** School of Anatomy, Physiology and Human Biology, The University of Western Australia, 35 Stirling Highway, Crawley, WA 6009 Australia; Stanford Partnership for Spinal Cord Injury and Repair, Department of Neurosurgery, School of Medicine, Stanford University, Lorry I. Lokey Stem Cell Research Building (SIM1), 265 Campus Drive, Stanford, CA 94305-5454 USA

## Erratum

After publication of [1] we became aware that not all of the authors revisions had been incorporated into the final PDF. The Publisher would like to sincerely apologize for this.

The following corrections should be made:

1. In the Background the phrase “However many previously discovered molecular cues in the mammalian visual system are diffusible, and the only vertebrate secreted members of the **Semaphorins** are the Class 3 Semaphorins (Sema3s)” should read “However many previously discovered molecular cues in the mammalian visual system are diffusible, and the only vertebrate secreted members of the **Semas** are the Class 3 Semaphorins (Sema3s)”
2. In the Results the phrase “Sema3a RNA levels were highly variable at E16 (149% coefficient of variance; CV), peaked at E19, and **a decline**” should read “Sema3a RNA levels were highly variable at E16 (149% coefficient of variance; CV), peaked at E19, and **declined**”
3. The Results subheading “Growth cone collapse assay for purified neonatal **RGCS***in vitro*” should read “Growth cone collapse assay for purified neonatal **retinal ganglion cells***in vitro”*
4. In the Results the phrase “these were not conducted at a developmental time point **where** the RGC axons are growing into and within their Sema3 expressing targets” should read “these were not conducted at a developmental time point **when** the RGC axons are growing into and within their Sema3 expressing targets”
5. In the Results the sentence “This difference in RNA expression levels might indicate that if **these** molecules are having an effect then the more likely candidates are Sema3c and Sema3f” should read “This difference in RNA expression levels might indicate that if **this family of** molecules are having an effect then the more likely candidates are Sema3c and Sema3f”
6. The sentence in the Conclusions “These new data, providing a framework for future studies aimed at elucidating such roles of the Sema3s.” should be removed.
7. The correct version of Additional File 2 is attached.
