# Supplementary figures and images for: Changes in expression of Class 3 Semaphorins and their receptors during development of the rat retina and superior colliculus
Source: BMC Dev Biol. 2014 Jul 26;14:34. doi: 10.1186/s12861-014-0034-9 (PMC4121511; doi:10.1186/s12861-014-0034-9)

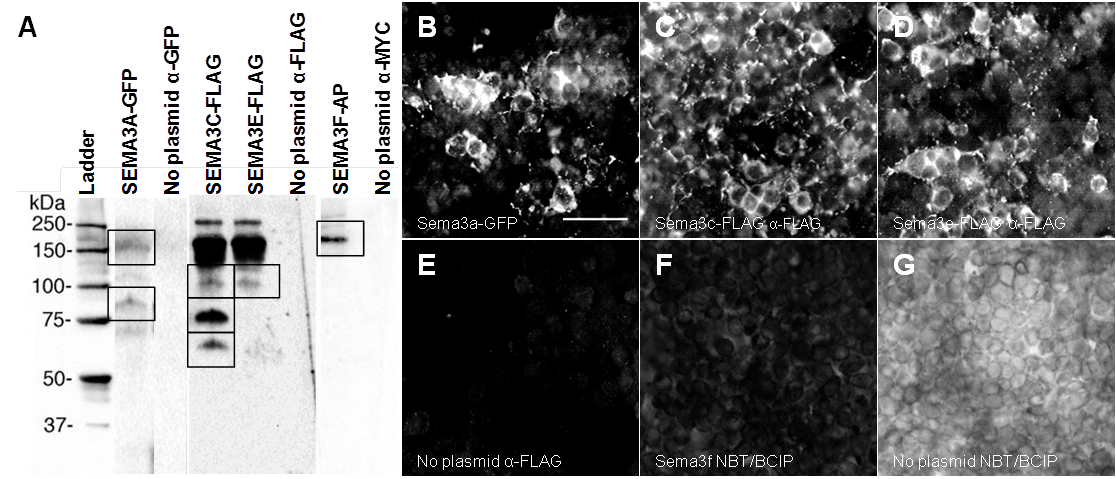

Supplement: Additional file 2: Figure S1. — Recombinant Sema3 protein expression in conditioned media. A: Presented are three different blots, lined up against the same ladder. Controls were from cultures that underwent a sham transfection (no plasmid). Boxes indicated bands of expected molecular weights. Detected bands for SEMA3A-GFP correspond to the approximately 130 kDa and 90 kDa bands reported previously [75]. SEMA3C-FLAG was detected at around 80 kDa as expected, and also in a presumably processed form at around 70 kDa. Both SEMA3C-FLAG and SEMA3E-FLAG were detected in media at approximately 100 kDa, which might represent glycosylated forms of the proteins. SEMA3F-AP was detected at 150 kDa, in line with expectations. B-G: recombinant Class 3 Semaphorin expression in HEK-293 T cells transfected with expression plasmids for Sema3a-GFP, Sema3c-FLAG, Sema3e-FLAG, and Sema3f-AP, as well as a no plasmid control. SEMA3A-GFP expression was visualised directly by epifluorescence (B), SEMA3B-FLAG (C) and SEMA3E-FLAG (D) was detected following fluorescence immunocytochemistry, and SEMA3F-AP was detected by the presence of colour reaction product from NBT/BCIP (F). Scale bar: 50 μM. [file s12861-014-0034-9-S2.tiff]
